# IFIT3 accelerates the progression of head and neck squamous cell carcinoma by targeting PD-L1 to activate PI3K/AKT signaling pathway

**DOI:** 10.1186/s12957-023-03274-5

**Published:** 2024-01-25

**Authors:** Peng Liu, Xin Kong, Shijiang Yi, Ying Chen, Wenlong Luo

**Affiliations:** 1 Department of Otolaryngology Head and Neck Surgery, the Second Affiliated Hospital of Chongqing Medical University, Chongqing, 400010 China; 2 Department of Infectious Diseases, Key Laboratory of Molecular Biology for Infectious Diseases, Institute for Viral Hepatitis, the Second Affiliated Hospital of Chongqing Medical University, Chongqing, China; 3 Department of Otolaryngology Head and Neck Surgery, the Affiliated Hospital of Guilin Medical University, Guilin, China; 4 Department of Traditional Chinese Medicine, the Affiliated Hospital of Guilin Medical University, Guilin, China

**Keywords:** IFIT3, PI3K/AKT pathway, PD-L1, EMT, CSCs

## Abstract

**Background:**

Emerging evidence has shown interferon-induced protein with tetratricopeptide repeats 3 (IFIT3) may be predicted to be a candidate oncogene and involved in the onset and progression of cancer, but IFIT3’s potential role in cancer, particularly in head and neck squamous cell carcinoma (HNSC), is not well recognized. This study aims to reveal the role of IFIT3 in HNSC and the underlying molecular mechanism.

**Methods:**

Bioinformatics analysis, immunohistochemical staining, RT-PCR, and Western blotting analysis were used to detect IFIT3 expression in HNSC. CCK-8 assays, colony formation assays, wound-healing assays, transwell assays, and sphere formation were used to explore proliferative, migratory, and invasive activities and cancer stemness of HNSC cells after IFIT3 knockdown and over-expressed. The alterations of EMT markers and PI3K/AKT pathway were detected by Western blotting. Animal studies were performed to analyze the effect of IFIT3 on tumor growth and metastasis of HNSC in vivo.

**Results:**

In this study, we observed that IFIT3 was highly expressed in HNSC, and its higher expression contributed to poorer survival of patients with clinical stage IV or grade 3. Function assay indicated that IFIT3 promoted malignant behaviors in vitro, as well as tumor growth and lung metastasis in vivo. Meanwhile, PD-L1 knockdown or over-expressed reversed cancer cell stemness, migration, invasion, and PI3K/AKT signaling pathway which were regulated by IFIT3.

**Conclusions:**

Our results reveal that IFIT3 promotes EMT and cancer stemness by targeting PD-L1 to activate PI3K/AKT signaling pathway in HNSC, and targeting IFIT3 may be a novel strategy for the treatment of patients with HNSC.

**Supplementary Information:**

The online version contains supplementary material available at 10.1186/s12957-023-03274-5.

## Introduction

Head and neck squamous cell carcinoma (HNSC) is one of the most common malignant tumors worldwide, with over 890,000 patients diagnosed and more than 450,000 deaths annually, accounting for 3% of all cancers [1]. Most HNSC patients are first diagnosed in the locally advanced stages and have regional lymph node (LN) metastases [2]. At present, the treatment of HNSC is either surgery or radiation, with or without chemotherapy; we also have made substantial progress in many promising and innovative combinatorial approaches, such as HPV vaccines, T-cell-directed therapies, and immunotherapy with immune-checkpoint inhibitors, but due to a high risk of local recurrence and distant metastasis, the 5-year survival rate for patients with advanced HNSC is still less than 50% [1, 3]. Therefore, it is particularly urgent to uncover the molecular mechanisms of HNSC progression that may provide insights into developing new, effective therapeutic strategies.

Interferon-induced protein with tetratricopeptide repeats 3 (IFIT3) belongs to the IFIT family proteins inside the cytoplasm and has been extensively examined for their antiviral characteristics, IFIT family consists of four members (IFIT1, IFIT2, IFIT3/4, and IFIT5), they have no known enzymatic activity, but they all contain unique structural patterns called tetratricopeptide repeats (TPRs), the TPRs serve as a structural component of IFIT proteins, consisting of 3 to 16 repeated and altered tandem sequences comprising 34 amino acids each, these TPRs are organized into helix-turn-helix configurations, and facilitating their participation in protein-protein interactions results in several protein complexes which play an important role in a variety of biological processes in cells [4]. Increasing evidence shows that IFIT family proteins play important roles in a variety of biological processes including double-stranded RNA signaling, viral replication, cell migration, and proliferation [5]. Some of these family members are predicted to be a candidate oncogene and involved in the onset and progression of cancer, including breast, pancreatic, lung, liver, and colorectal cancers, as well as renal cancer playing key roles in epithelial-mesenchymal transition (EMT) [6–8]. EMT is crucial for promoting tumor cell migration, invasion, metastasis, malignant transformation, and tumor stemness, and cancer stem cells (CSCs) constitute a unique subset of tumor cells characterized by enhancing tumorigenic potential, self-renewal abilities, and the capacity to differentiate [9]. Therefore, explaining and discovering new mechanisms by which EMT and CSCs regulate cancer progression may be an effective way to improve HNSC treatment. Early studies demonstrate that abnormal expression of IFIT3 is associated with lupus erythematosus and systemic lupus erythematosus [10]. IFIT3 plays crucial role in antiviral innate immunity and has been involved in cell proliferation, apoptosis, differentiation, and cancer development [11]. However, the effects of IFIT3 on cancer, particularly in HNSC, and its molecular mechanisms remain undefined. Further studies are needed to clarify how IFIT3 regulates HNSC carcinogenesis.

The function of PI3K/AKT signaling pathway has been widely reported in cellular biological process, highlighting its crucial role in cancer development and progression, such as cancer cell proliferation, differentiation, metabolism, angiogenesis, migration, and invasion [12]. Recently, programmed death-ligand 1 (PD-L1) is found to be involved in the regulation of PI3K/AKT pathway, the multifaceted functions of PD-L1 beyond its well-known role in immune suppression of T cells, such as promoting EMT and sustaining cancer stemness [13]. Therefore, based on bioinformatics analysis, we explore the relationship between them. In this study, we found that IFIT3 was upregulated in HNSC. Function assay demonstrated IFIT3 promoted cell proliferation, migration, and invasion. Further, detection of molecular mechanisms revealed that IFIT3 enhanced EMT processes and CSCs properties by targeting PD-L1 to activate PI3K/Akt pathway in HNSC. Our results provide novel insights into the role and underlying molecular mechanism of IFIT3 in HNSC.

## Methods

### Data collection and processing

TCGA pan-cancer datasets were downloaded from https://portal.gdc.cancer.gov/. Meanwhile, we collected 15 pairs of HNSC and adjacent tumor tissue samples from GSE58911 dataset which was downloaded from GEO database (http://www.ncbi.nlm.nih). We used limma package in R software (version 3.6.4) to compare the difference of IFIT3 expression between tumor samples and normal samples. Kaplan–Meier plotter database (https://kmplot.com/analysis/) was used to analyze the relationship between IFIT3 expression and prognosis.

### Clinical samples

One-hundred one surgically resected primary HNSC tissue specimens and 57 of these patients who had paired adjacent non-tumorous tissues were collected from the Department of Otolaryngology Head and Neck Surgery at the Affiliated Hospital of Guilin Medical University. They were used for the construction of tissue microarrays for immunohistochemical (IHC) staining. All patients were first pathologically diagnosed as squamous cell carcinoma, and all patients did not receive any anticancer treatment prior to biopsy. Signed informed consent was obtained from all patients, and the study was approved by the Institutional Ethics Committee of the Second Affiliated Hospital of Chongqing Medical University and the Affiliated Hospital of Guilin Medical University.

### Cell culture

Human HNSC cell lines (FaDu and Cal27) were purchased from Procell Life Science and Technology Co. Ltd. (Wuhan, China). FaDu cells were cultured in MEM medium (Gibco, USA), and Cal27 cells were cultured in DMEM medium (Gibco, USA). All cultures were supplemented with 1% penicillin-streptomycin (Solarbio, China) and 10% fetal bovine serum (Clark, USA). All cells were cultured in incubators at 37 °C and 5% CO_2_.

### Immunohistochemistry (IHC) staining

IHC staining was conducted on tissue microarray following a standard staining procedure as the universal two-step detection kit (mouse/rabbit-enhanced polymer detection system) (ZSGB-BIO, China) described. In brief, paraffinization and rehydration were performed, following antigen retrieval, tissue microarray was treated with 3% hydrogen peroxide for the blocking of endogenous peroxidases, and then 1:100 diluted IFIT3 antibody (HPA059914, Atlas Antibodies), 1:100 diluted PD-L1 antibody (CST, USA), and 1:100 diluted ki67 antibody (Affinity, China) were used to incubate tissue sections overnight at 4 °C. After washing, incubation was conducted for 1 h at room temperature with secondary antibodies biotinylated with goat anti-rabbit IgG. A chromogen was prepared using diaminobenzidine, and the slide was then counterstained with hematoxylin. The product of percentage of positive stained cells and the staining intensity scores were calculated to evaluate expression. We graded staining intensity according to four criteria: 0, no staining; 1, yellow; 2, deep yellow; and 3, brown. A proportion of stained cells was scored as follows: 0 (0–10%), 1 (10–25%), 2 (25–50%), 3 (50–75%), or 4 (75–100%). The median of total scores was used as the cutoff point to discriminate a high-expression group and a low-expression group.

### Cell transfection

Lentiviral vectors containing short hairpin RNAs (shRNAs) targeting IFIT3 (shIFIT3#1 and shIFIT3#2), control vectors (shNC), PD-L1 over-expression of lentiviral vector (OE-PD-L1), and an empty control lentiviral vector (VEC) were obtained from GeneChem Company (Shanghai, China). IFIT3 over-expression lentiviral vector (OE-IFIT3) and an empty control lentiviral vector (Vector), shRNA targeting PD-L1 (shPD-L1) and negative control (shcon), were purchased from Tsingke Biotechnology Co., Ltd. (Beijing, China). Following the manufacturer’s instructions, HiTransG P was employed to encourage transfection, and those lentiviral vectors were then added to infect FaDu and Cal27 cells at an multiplicity of infection (MOI) of 20. Infection efficiency was evaluated by qRT-PCR and Western blot. The shRNA sequences are listed in Table S1.

### Quantitative real-time PCR

RNAsimple total RNA Kit (TIANGEN, China) was used to extract RNA from cell lines and HNSC tissues, and 1 µg of total RNA was reverse transcribed by FastKing gDNA Dispelling RT SuperMix (TIANGEN, China) and amplifed by QuantStudio Real-Time PCR system (Thermo Fisher Scientific, Inc., USA) with RealUniversal color premix (SYBR green) (TIANGEN, China). GAPDH was selected as an internal control, and the relative expression was analyzed using the 2^−△△Ct^ method. Primer sequences are listed in Table S1.

### Cell Counting Kit-8 (CCK-8) assay

CCK-8 kit (Life-iLab, China) was used to assess cell viability. A total of 2000 transfected FaDu and Cal27 cells (shNC, shIFIT3#1, shIFIT3#2, vector, and OE-IFIT3) in 100 μl of medium were plated into each well of 96-well microplates. After culture for 1, 2, 3, and 4 days, following the addition of CCK-8 reagent (10 μl per well), the cells were cultured at 37 °C in the dark for another 2 h and measured at a wavelength of 450 nm via microplate reader (Thermo Fisher Scientific, Inc., USA).

### Colony formation assay

The transfected FaDu and Cal27 cells were digested with 0.25% trypsin, suspended in complete medium, and then counted. A total of 1000 cells were inoculated in each well and incubated at 37 °C with 5% CO_2_ in a humidified incubator for 14 days. When the cells formed visible colonies and gently washed with PBS, the colonies were fixed using 4% paraformaldehyde for 20 min and stained with 0.1% crystal violet after washing with PBS. Then, the colonies were rinsed using distilled water, dried and photographed, and finally quantified under a microscope.

### Isolation of CD44+ cells and flow cytometry

CD44+ cells were isolated from FaDu and Cal27 cells using the CD44 MicroBeads (Miltenyi Biotec, Germany). After magnetic labeling and magnetic separation with LS columns and LD columns, cells were then sorted by using MidiMACS^™^ separator (Miltenyi Biotec, Germany), stained with CD44-PE antibody (CST, USA), and analyzed by flow cytometry (BD Biosciences, USA). The stably transfected FaDu and Cal27 cells were collected and resuspended in 100 μl of phosphate-buffered saline (PBS) containing 20 μl FcR blocking reagent (Miltenyi Biotec, Germany), cells were also stained with CD44 antibody for 15 min in the dark, and CD44 expression was detected by flow cytometry.

### Tumor sphere formation assay

A total of 1000 cells/per well were seeded in an ultralow attachment 96-well plate (Corning, USA) and cultivated with serum-free DMEM/F12 medium (Gibco, USA) containing 20 ng/ml EGF (MedChemExpress, USA), 10 ng/ml b-FGF (MedChemExpress, USA), and 2% B27 (MedChemExpress, USA). After incubation for 10 days, the images of spheres were captured under a microscope.

### Wound‑healing assay

Until the suitably transfected cells were almost 100% confluence after 24 h of culture, we used a 200 μl sterile pipette tip to scratch through the confluent monolayers, then an artificial blank area was created on the fused monolayer cells, called the “wound area,” and the injured monolayer cells were washed with PBS to remove cellular debris. Subsequently, the cells were maintained in a serum-free medium for 48 h. Wound area was photographed with microscopy at 0 and 48 h, the cell coverage area was evaluated using Image J, and the wound closure rate was calculated using the following formula:

$$\mathrm{Wound~closure~rate}~(\mathrm{\%}) = [1-(\mathrm{Wound~area~at }~48\mathrm{~h}/\mathrm{Wound~area~at}~ 0\mathrm{~h})] \times 100\mathrm{\%}$$

### Transwell assays for migration and invasion

A 24 well transwell chambers (8 μm pore, BD Biosciences, USA) were coated with or without 0.2% Matrigel (BD Biosciences, USA). A total of 2.5 × 10^4^ transfected cells in 150 μl cell medium without FBS were added to the upper chamber, and the lower chamber was filled with a total of 700 μl of culture media containing 10% FBS. After incubation for 24 h, using a cotton swab, the non-invading cells on the upper chamber were delicately eliminated. The cells that migrated or invaded to the lower chamber were fixed with 4% paraformaldehyde for 15 min and stained with 0.1% crystal violet for 20 min. The cells in five random fields were photographed and counted under a microscope.

### Functional enrichment analysis

TCGA HNSC cohort were divided into high-expression group and low-expression group based on the median of IFIT3 expression. Kyoto Encyclopedia of Genes and Genomes (KEGG) enrichment analysis was performed by the ClusterProfiler R package. We also collected the publicly available “hallmark” related signature and reactome gene sets from the molecular signatures database (MSigDB). Gene set enrichment analysis (GSEA) on each pathway was calculated in order to obtain the correlation between the sample and the pathway.

### Western blotting analysis

Tissue samples and transfected cell lines were lysed with RIPA lysis buffer containing 0.1 mg/ml PMSF (Solarbio, China) for 30 min on ice. After centrifuging at 12,000 g for 15 min at 4 °C, the supernatant protein concentrations were determined by BCA assay kit (Beyotime, China) and denatured. Next, the proteins were then separated by 10% sodium dodecyl sulfate-polyacrylamide gel electrophoresis and transferred onto 0.45 μm polyvinylidene difluoride membranes (Millipore, USA).

After blocking with QuickBlock^™^ blocking buffer for Western blot (Beyotime, China) for 20 min at room temperature, the membranes were incubated with appropriate primary antibodies overnight at 4 °C, followed by incubation with the secondary antibodies for 1.5 h at room temperature. The protein bands were treated with the EZ ECL pico luminescence reagent(Life-iLab, China) and visualized by the Molecular Imager ChemiDoc XRS System (Bio-Rad, USA). The primary and secondary antibodies are shown in Table S2.

### Co-immunoprecipitation (Co-IP)

FaDu and Cal27 cells were washed with PBS and lysed by cell lysis buffer for western and IP (Beyotime, China), and 2-μg antibody and control IgG were added to the cell lysate and slowly shaken at 4 °C overnight. Then, this mixture was incubated with protein A+G agarose (Beyotime, China) at 4 °C for 3 h. After centrifugation, the supernatant was discarded, and immunoprecipitation was washed with PBS, then the immunoprecipitation was resuspended in loading buffer and incubated at 100℃ for 5min, the interacting protein was detected by Western blotting analysis.

### Immunofluorescence (IF) assay

FaDu and Cal27 cells were seeded in cell climbing films at appropriate density. Cells were fixed using 4% paraformaldehyde for 10 min, incubated with 0.1% Triton X-100 in PBS for 10 min, and treated with blocking solution containing 10% goat serum for 1 h at room temperature. Primary antibodies (IFIT3, PD-L1) were added into the cell climbing films and incubated at 4 °C overnight in a dark humidity chamber. The samples were incubated in secondary antibodies in a dark humidity chamber at room temperature for 1 h and then mounted using mounting medium antifading (with DAPI). Fluorescence images were taken by Zeiss laser confocal fluorescence microscope.

### In vivo xenograft model and metastasis mode

The animal experiment protocol was approved by the Animal Care Welfare Committee of Chongqing and Guilin Medical University. Twenty-four male BALB/c nude mice aged 4 to 6 weeks (Hunan SJA Laboratory Animal Co., Ltd.) were randomly divided into four groups: shNC, shIFIT3#1, Vector, and OE-IFIT3, to establish xenograft model, a total of 5 × 10^6^ transfected Cal27 cells suspended in 200 μl PBS was injected in the right axilla of nude mice, and tumor volume was determined by using the following formula: volume = length × width^2^/2. Twelve male BALB/c nude mice aged 4 to 6 weeks were randomly divided into four groups: shNC, shIFIT3#1, Vector, and OE-IFIT3, a total of 2.5 × 10^6^ transfected Cal27 cells suspended in 100 μl PBS was injected via the tail vein of nude mice to establish metastasis models, and their lung tissues were fixed and stained with H&E staining. All the mice were sacrificed after 5 weeks.

### Statistical analysis

GraphPad Prism 8 and R software (version 3.6.4) were used to conduct all statistical analyses. Mann-Whitney *U *tests were used to compare the IFIT3 expression levels in TCGA and GEO datasets. Chi-square tests were performed to confirm the relationship between IFIT3 expression levels and clinicopathological features. Continuous data was applied by Student’s tests or one-way ANOVA to compare group pairs and multiple groups; the statistical results were expressed as the mean ± standard deviation from three independent experiments. *P* < 0.05 was considered statistically significant, and the significance was indicated by *P < 0.05, **P < 0.01, ***P < 0.001, and ****P < 0.0001.

## Results

### IFIT3 expression was over-expressed in HNSC

**Fig. 1:**
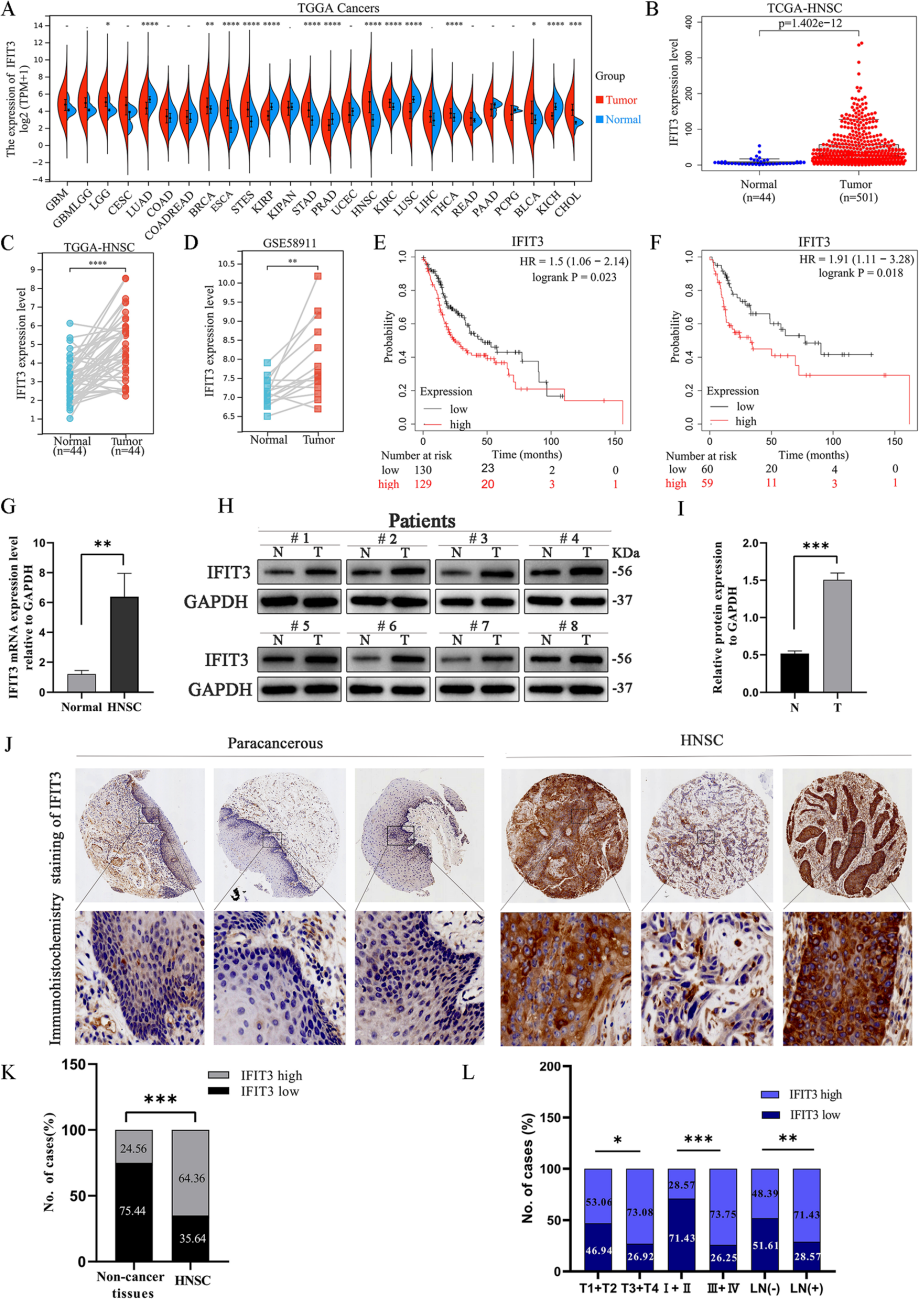
Expression of IFIT3 in HNSC. **A** The expression of IFIT3 in TCGA pan-cancer database. **B** The expression of IFIT3 in TCGA HNSC database. **C** The expression of IFIT3 in tumor tissues and matched normal tissues in TCGA HNSC. **D** The expression of IFIT3 in tumor tissues and matched normal tissues in GSE58911. **E** Kaplan–Meier curves of the overall survival rate of patients with clinical stage IV. **F** Kaplan–Meier curves of the overall survival rate of patients with grade 3. **G** The IFIT3 mRNA levels in 20 pairs of HNSC and adjacent normal tissues were analyzed by RT-PCR. **H** and **I** The IFIT3 protein levels in eight pairs of HNSC and adjacent normal tissues were analyzed by Western blotting. **J** Representative images of IHC analysis for IFIT3 expression in HNSC. **K** IFIT3 high expression in HNSC. **L** The clinical significance of IFIT3 expression

IFIT3 expression levels were analyzed from the TCGA database. It was increased in the majority of the cancer types including HNSC (*P* < 0.0001), LGG, BRCA, ESCA, and STES (Fig. 1A). High expression of IFIT3 was found in HNSC (Fig. 1B); it was also significantly highly expressed in 44 cases of HNSC tumor than matched adjacent normal tissue samples (*P* < 0.0001) (Fig. 1C). GSE58911 dataset also showed a higher IFIT3 expression in HNSC tissues than that in adjacent normal tissues (*P* < 0.05) (Fig. 1D). The Kaplan-Meier plotter was used to explore the associations between IFIT3 expression and the overall survival (OS) in TCGA HNSC cohort, we found that high IFIT3 expression had worse overall outcomes in patients with clinical stage IV (*HR* = 1.5, *P* < 0.05) (Fig. 1E) or poorly differentiated (grade 3) (*HR* = 1.91, *P* < 0.05) (Fig. 1F). Up-regulation of IFIT3 was further validated by real-time PCR (Fig. 1G) and Western blotting analysis (Fig. 1H and I). Therefore, the findings suggested that IFIT3 may act as an oncogene in HNSC.

In order to investigate the relationship between IFIT3 expression and clinicopathological features of HNSC patients, we performed IHC analysis to detect the IFIT3 expression on the HNSC tissue microarray containing 101 tumor tissues and 57 adjacent normal tissues. IHC analysis showed that IFIT3 was mainly localized in the cytoplasm (Fig. 1J). High expression of IFIT3 was observed in 65 out of 101 (64.36%) HNSC tissues but in only 14 out of 57 (24.56%) adjacent normal tissues (*P* < 0.001) (Fig. 1K). As summarized in Table 1, there was no significant correlation between IFIT3 expression and age or tumor grade (*P* > 0.05), while high IFIT3 expression was significantly correlated with the pathological T classification, clinical stage, and lymph node (LN) metastasis (*P* < 0.05) (Fig. 1L). Collectively, these results implied that high IFIT3 expression may be a drive event and contribute to HNSC progression.

**Table 1:** Correlation of IFIT3 expression with the clinicopathological characteristics of HNSC

Characteristics	Total	IFIT3 expression		${\chi }^{2}$	*p*-value
		Low	High		
Age (y)					
< 60	47	21	26	3.13	0.077
≥ 60	54	15	39		
T classification					
T1+T2	49	23	26	4.35	0.037
T3+T4	52	14	38		
Tumor grade					
1	38	13	25	5.56	0.062
2	51	15	36		
3	12	8	4		
Clinical stage					
I + II	21	15	6	14.80	0.000
III + IV	80	21	59		
Lymph node metastasis					
No	31	16	15	4.97	0.026
Yes	70	20	50		

### IFIT3 facilitated the proliferative and maintained cell stemness in HNSC cells

**Fig. 2:**
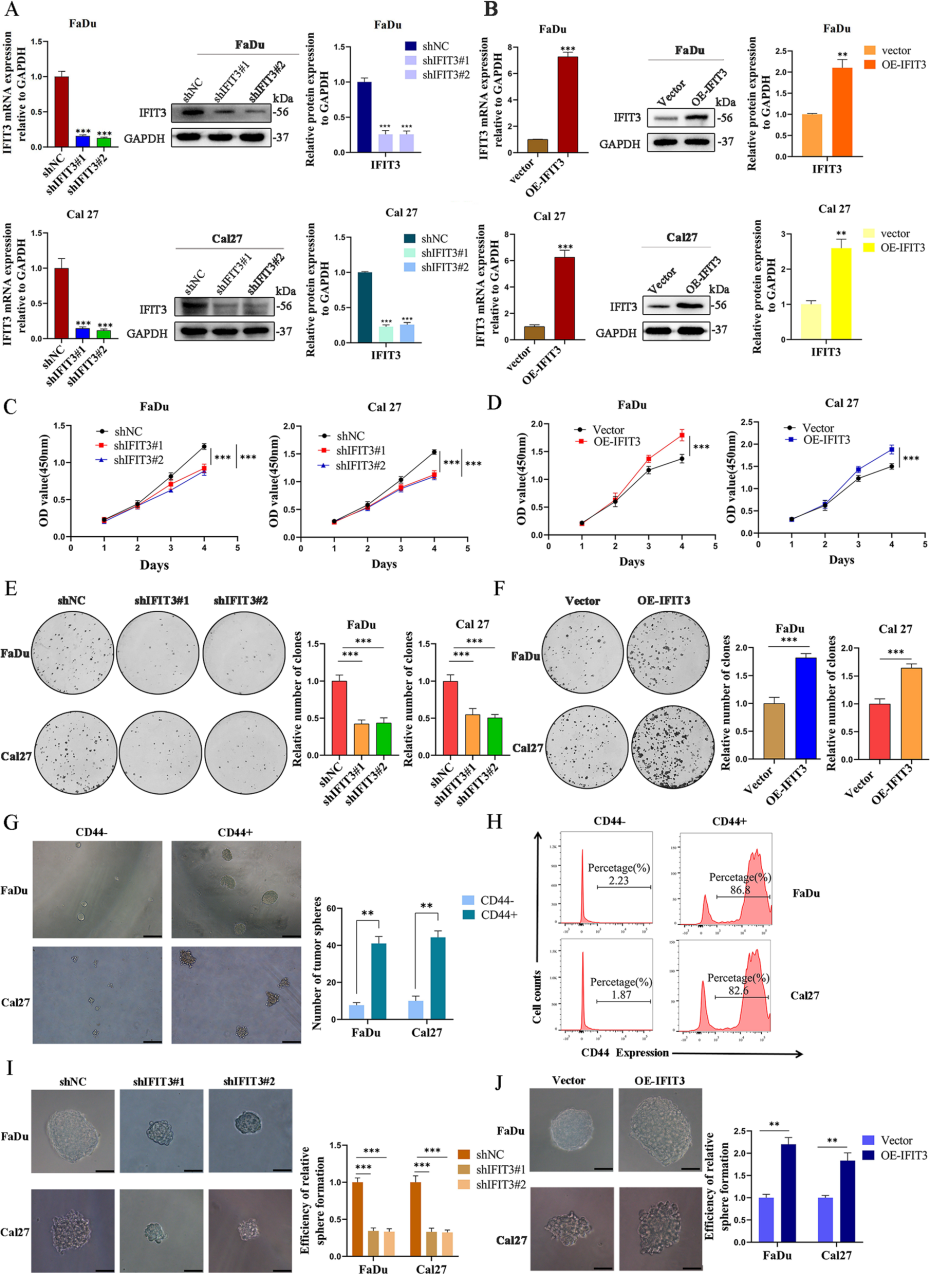
IFIT3 promoted HNSC cell growth and cell stemness in vitro. **A** Verification of transfection efficiency of IFIT3 knockdown in FaDu and Cal 27 cells at mRNA and protein levels. **B** Verification of transfection efficiency of IFIT3 up-regulation in FaDu and Cal 27 cells at mRNA and protein levels. **C** The viability of IFIT3 knockdown in FaDu and Cal 27 cells was assessed via CCK-8 assay. **D** The viability of IFIT3 up-regulation in FaDu and Cal 27 cells was assessed via CCK-8 assay. **E** The impact of IFIT3 knockdown on the clone formation ability in FaDu and Cal 27 cells. **F** The impact of IFIT3 up-regulation on the clone formation ability in FaDu and Cal 27 cells. **G** Representative images of CD44^+^ and CD44^−^ cells isolated from FaDu and Cal 27 cells after 10 days in serum-free culture media. Scale bar, 200 μm. **H** Flow cytometry detected the distribution of CD44^+^ cells. Scale bar, 200 μm. **I** The sphere formation ability of IFIT3 knockdown in FaDu and Cal 27 cells. Scale bar, 200 μm. **J** The sphere formation ability of IFIT3 up-regulation in FaDu and Cal 27 cells. Scale bar, 200 μm

To clarify whether IFIT3 functioned in HNSC behavior, we knocked down and over-expressed IFIT3 in FaDu and Cal27 cells by transfection with lentiviral vectors. The transfection effectiveness was validated at mRNA and protein levels (Fig. 2A and B). When IFIT3 was knockdown, CCK8 assay (Fig. 2C and D) and colony formation assay (Fig. 2E and F) revealed that the viability of FaDu and Cal27 cells was inhibited, while IFIT3 up-regulation showed the opposite.

**Fig. 3:**
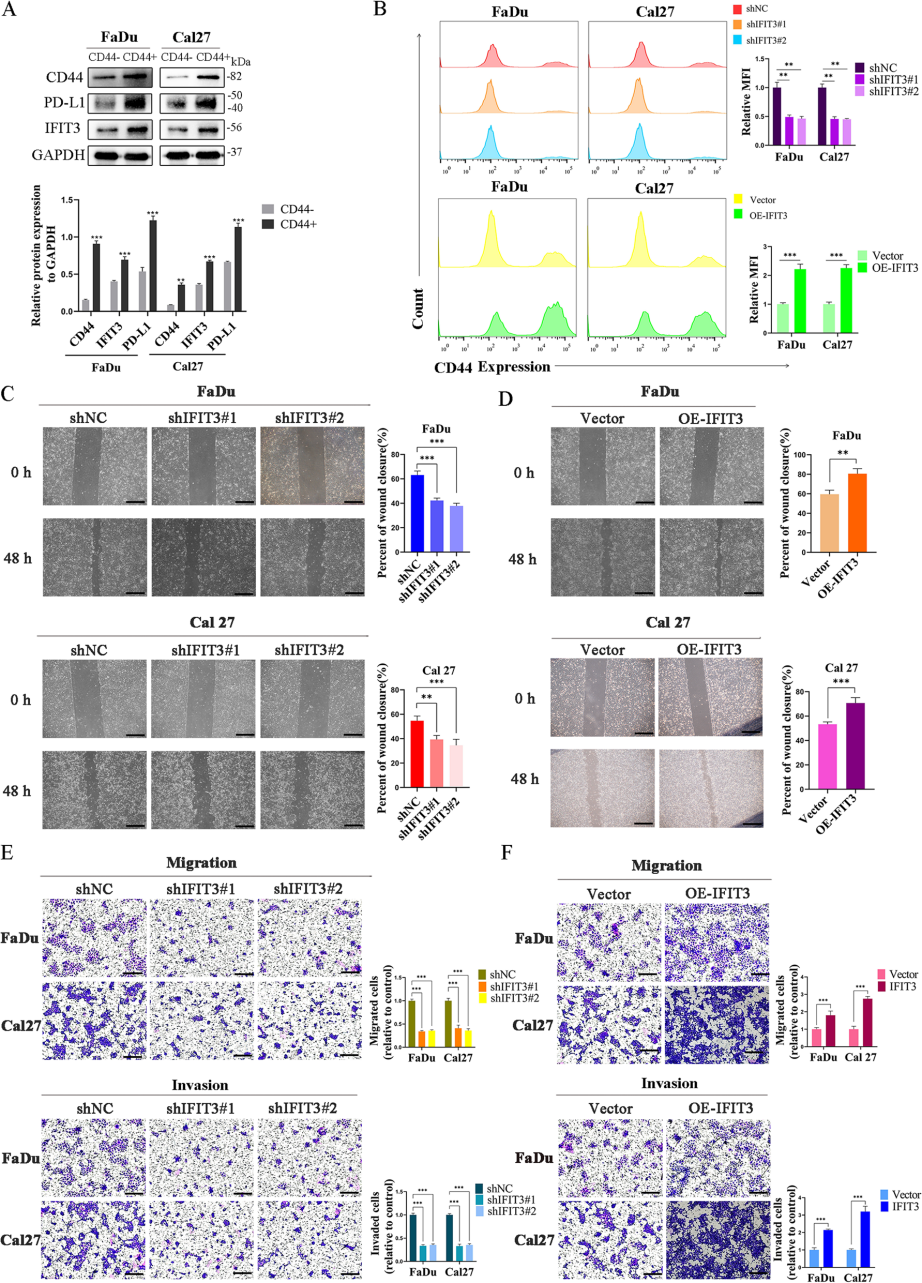
IFIT3 enhanced migration and invasion of HNSC cells. **A** CD44, IFIT3, and PD-L1 expression levels in CD44^+^ and CD44^−^ cells. **B** Flow cytometry of the distribution of CD44^+^ cells in IFIT3 knockdown and up-regulation in FaDu and Cal 27 cells. **C** Cells migration at 48 h after scratching in FaDu and Cal27 cells with IFIT3 knockdown were determined by wound-healing assays. Scale bar, 200 μm. **D** Cells migration at 48 h after scratching in FaDu and Cal27 cells with IFIT3 over-expression were determined by wound-healing assays. Scale bar, 200 μm. **E** The effect of IFIT3 knockdown on the migration and invasion of FaDu and Cal27 cells. Scale bar, 200 μm. **F** The effect of IFIT3 over-expression on the migration and invasion of FaDu and Cal27 cells. Scale bar, 200 μm

CD44 is a CSC marker conserved across cancer types including HNSC [14]. We sorted CD44^+^ cells from FaDu and Cal27 cells. The number of spheroids (diameter > 50 μm) in the CD44+ cells was notably more than that in the CD44^−^ cells (Fig. 2G). Flow cytometric analysis showed that the portion of CD44^+^ FaDu and Cal27 cells was more than 80% after sorting (Fig. 2H). Additionally, IFIT3 knockdown obviously suppressed the formation of spheroids in FaDu and Cal27 cells (Fig. 2I), and IFIT3 over-expression remarkably increased the formation of spheroids (Fig. 2J). Subsequently, Western blotting showed higher CD44, IFIT3, and PD-L1 expression in CD44^+^ cells formation of spheroids in comparison with CD44^−^ cells formation of spheroids (Fig. 3A). Compared to the control cells, knockdown IFIT3 expression in FaDu and Cal27 cells obviously decreased the portion of CD44^+^ cells and up-regulated IFIT3 expression in FaDu and Cal27 cells increased the portion of CD44^+^ cells (Fig. 3B).

### IFIT3 promoted migration and invasion in HNSC cells

Wound-healing assay, transwell migration, and invasion assay were used to assess the migration and invasion abilities of IFIT3 knockdown and over-expressed in FaDu and Cal27 cells. We found that the spreading range was decreased in the FaDu shIFIT3#1/2 and Cal27 shIFIT3#1/2 cells compared with the controls by the wound-healing assay (Fig. 3C); the results indicated that cells with higher IFIT3 expression showed significantly more rapid wound closure than controls (Fig. 3D). Additionally, cell mobility and invasive capability were dramatically attenuated when IFIT3 knockdown in FaDu and Cal27 cells, whereas cell mobility and invasive capability were enhanced when IFIT3 up-regulated in FaDu and Cal27 cells (Fig. 3E and F).

### IFIT3 accelerated EMT phenotype and activated PI3K/AKT signaling pathway in HNSC

**Fig. 4:**
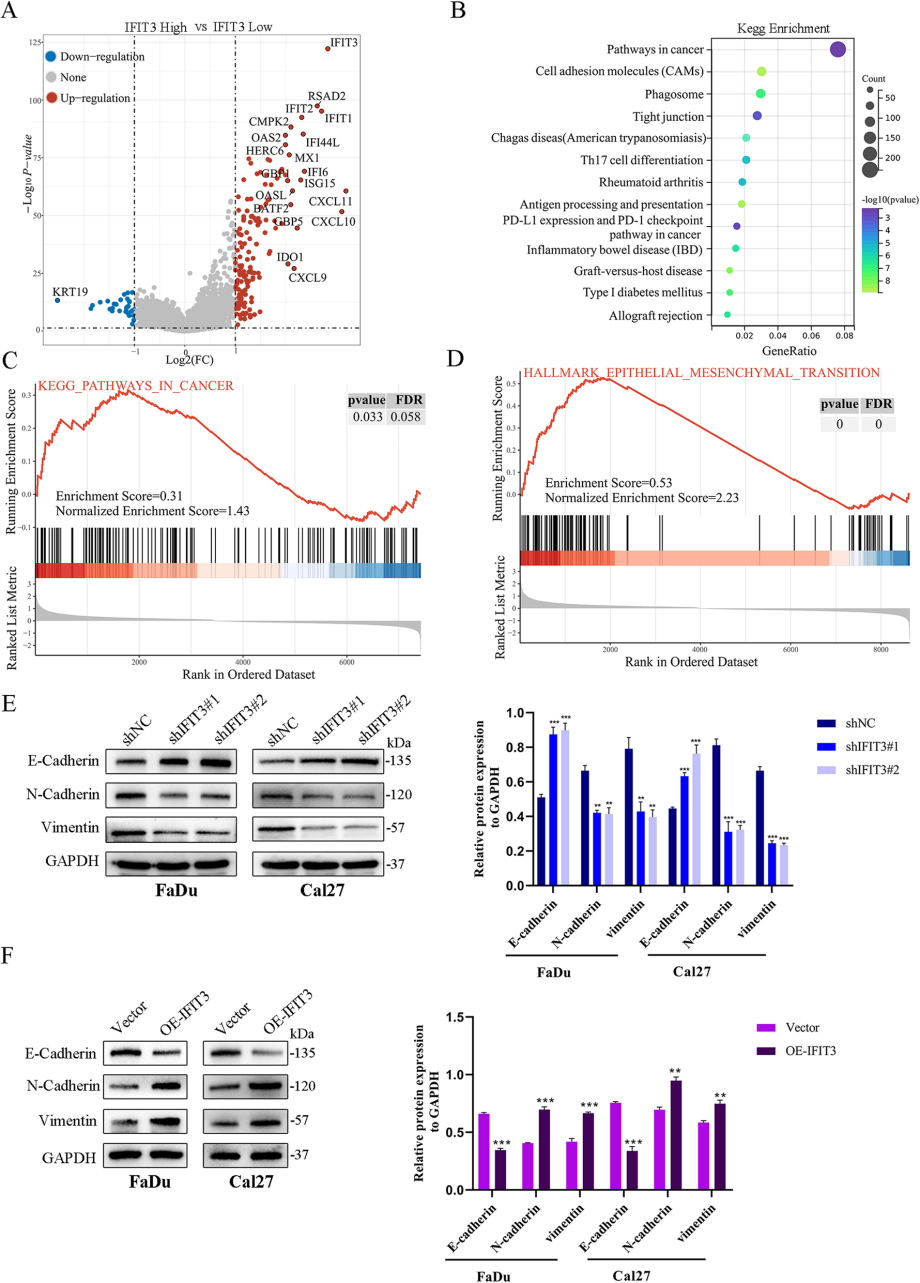
IFIT3 regulated the activation of EMT progress in HNSC cells. **A** The different expression genes between IFIT3 high groups and low groups. **B** KEGG pathway analysis of these different expression genes. **C** Enrichment analysis of pathways in cancer by GSEA. **D** Enrichment analysis of EMT by GSEA. **E** and **F** Protein levels of E-cadherin, N-cadherin, and vimentin were determined by Western blotting in IFIT3 knockdown and up-regulation in FaDu and Cal 27 cells

**Fig. 5:**
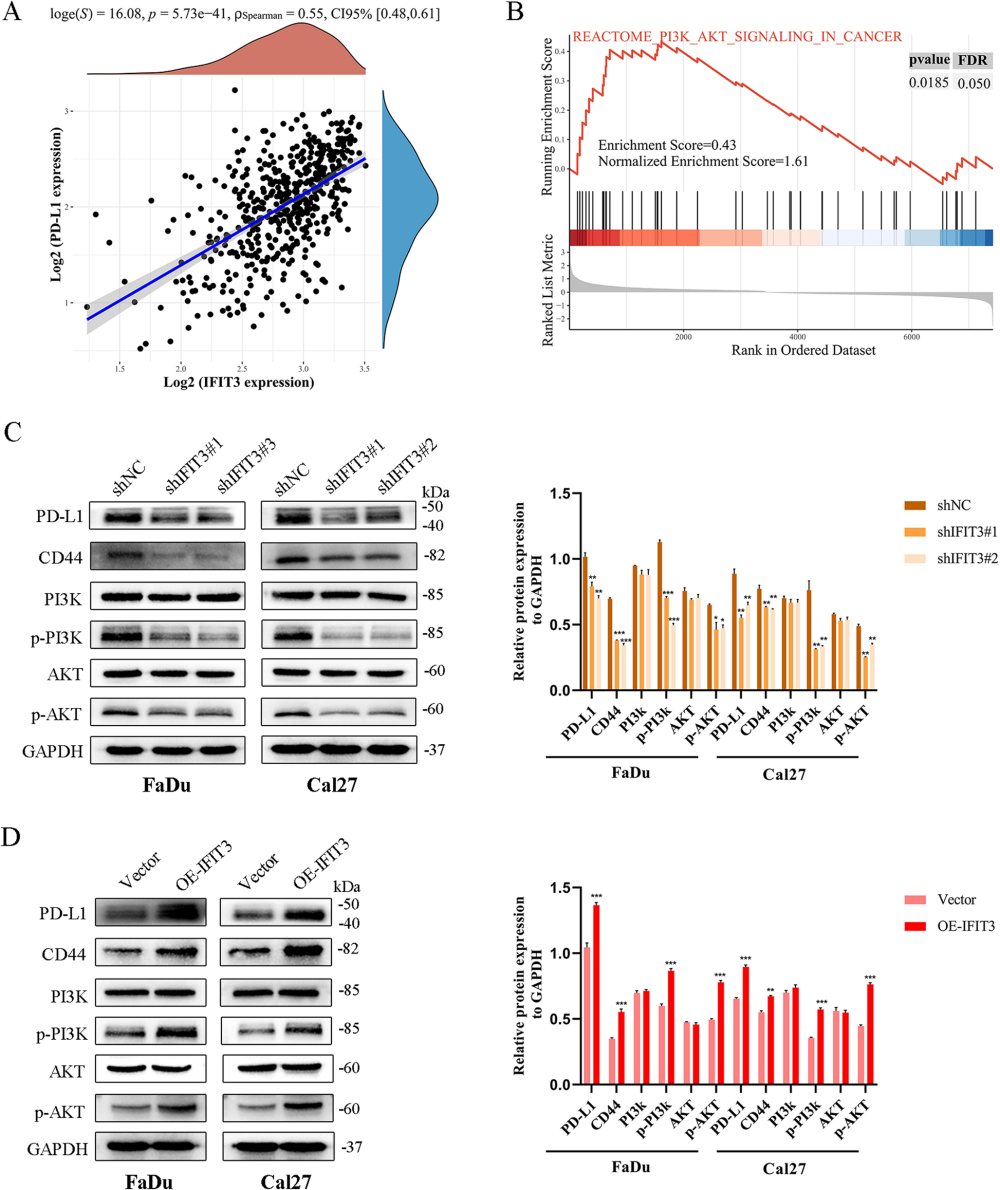
IFIT3 regulated the activation of PD-L1 and PI3K/AKT signaling pathway in HNSC cells. **A** PD-L1 mRNA level was positively correlated with IFIT3 in TCGA HNSC. **B** Enrichment analysis of PI3K/AKT pathway in cancer by GSEA. **C** and **D** CD44, PD-L1, PI3K, p-PI3K, AKT, and p-AKT protein levels were measured by Western blotting

To provide new insights into the potential functions and molecular mechanisms, we split TCGA HNSC samples into high-expression group and low-expression group based on the median of IFIT3 expression. The volcano plot depicted that different expression genes between the two groups (Fig. 4A). KEGG enrichment analyses showed they were involved in pathways in cancer, cell adhesion molecules, phagosome, tight junction, and PD-L1 expression and PD-1 checkpoint pathway in cancer, and so on (Fig. 4B). GSEA analysis of two groups revealed that the different expression genes in IFIT3 high-expression group were significant overlaps in pathways in cancer and EMT-related genes (Fig. 4C and D). We detected typical EMT markers by Western blotting. IFIT3 knockdown elevated the expression of E-cadherin and suppressed N-cadherin and vimentin expression in FaDu and Cal27 cells (Fig. 4E). In contrast, IFIT3 upregulation in FaDu and Cal27 cells could significantly reduce E-cadherin but increase N-cadherin and vimentin expression (Fig. 4F). Besides, we found that PD-L1 mRNA level was positively correlated with IFIT3 in TCGA HNSC, and GSEA analysis showed that the PI3K/AKT pathway was one of the important pathways involved (Fig. 5A and B). We demonstrated that IFIT3 knockdown suppressed PD-L1, CD44, the level of phosphorylated PI3K and AKT in FaDu and Cal27 cells, as expected, and IFIT3 up-regulation exerted the opposite effects (Fig. 5C and K). These findings indicated that IFIT3 played a vital role in the process of EMT and activated on the PI3K-AKT pathway.

### IFIT3 regulated EMT and CSCs by targeting PD-L1 through PI3K/AKT pathway in HNSC

**Fig. 6:**
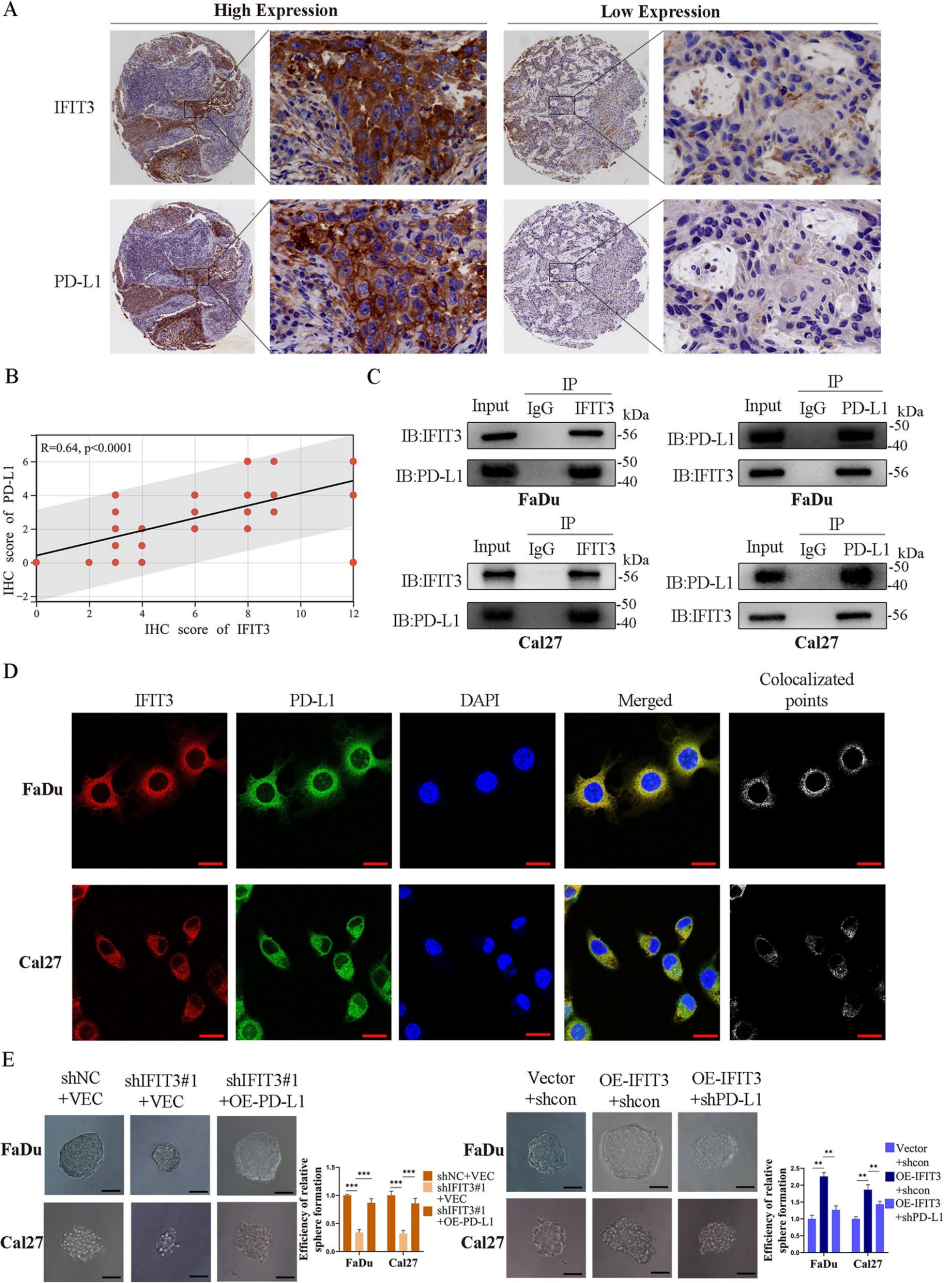
IFIT3 interacts with PD-L1 in HNSC. **A** and **B** IHC analysis showed PD-L1 expression was positively correlated with IFIT3. **C** IFIT3 protein interacting with PD-L1was examined in FaDu and Cal27 cells by co‑ip. **D** Co-localization based on IF assays, IFIT3 interacted with PD-L1 in the cytoplasm. Scale bar, 20 μm. **E** Sphere formation experiment was used to detect the effect of PD-L1 on sphere formation ability caused by IFIT3. Scale bar, 200 μm

**Fig. 7:**
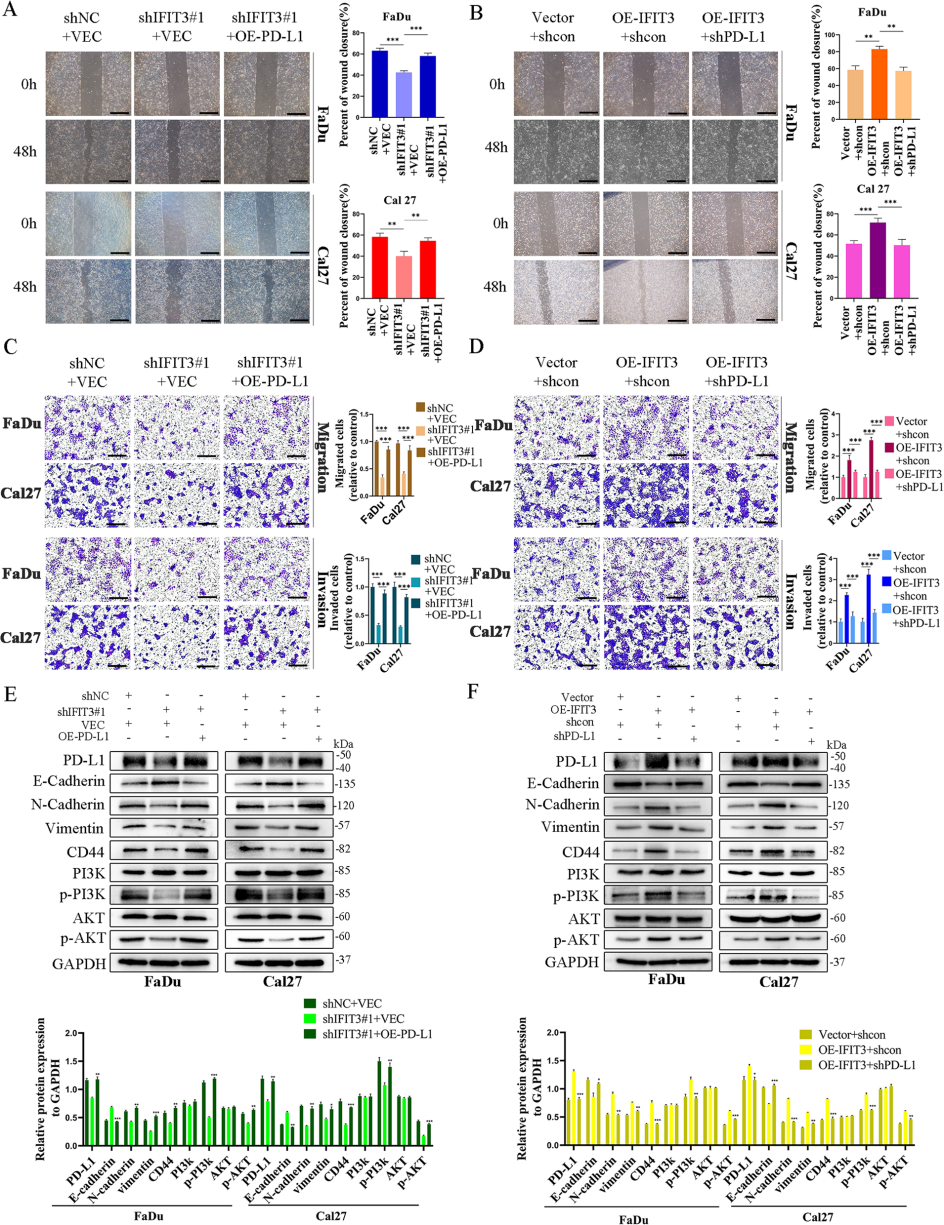
IFIT3 induced EMT and cancer cell stemness through PI3K/AKT pathway by targeting PD-L1 in HNSC. **A** PD-L1 over-expression dampened IFIT3 silencing suppressed migration by wound-healing assays. Scale bar, 200 μm. **B** PD-L1 knockdown dampened IFIT3 over-expression induced migration by wound-healing assays. Scale bar, 200 μm. **C** PD-L1 over-expression reversed IFIT3 silencing suppressed migration and invasion by transwell assays. Scale bar, 200 μm. **D** PD-L1 knockdown reversed IFIT3 over-expression induced migration and invasion by transwell assays. Scale bar, 200 μm. **E** and **F** Targeting PD-L1 attenuated IFIT3 induced EMT and CSCs markers protein expression levels in FaDu and Cal27 cells

6D-L1 has a critical role in tumor progression [15]. IHC analysis showed PD-L1 was positively correlated with IFIT3 in HNSC tissues (Fig. 6A and B). Then, we confirmed interaction between IFIT3 and PD-L1 in FaDu and Cal27 cells by co-ip assays (Fig. 6C). Meanwhile, the co-localization of the two proteins was in the cytoplasm of FaDu and Cal27 cells confirmed by IF assays (Fig. 6D). To investigate whether IFIT3 regulated EMT and CSCs by targeting PD-L1 in HNSC, stably knockdown IFIT3 in FaDu and Cal27 cells were co-transfected with over-expressed PD-L1 vector, PD-L1 up-regulation significantly abolished the inhibiting effect of IFIT3 downregulation on the formation of spheroids, migration, and invasion abilities in FaDu and Cal27 cells, and PD-L1 silencing blunted the promoting effect of IFIT3 over-expression on the formation of spheroids, migration, and invasion abilities in FaDu and Cal27 cells (Fig. 6E, 7A–D). We further examined whether IFIT3 activated PI3K-AKT signaling pathway through mediating PD-L1, and PD-L1 up-regulation reversed the expression of E-cadherin, vimentin, N-cadherin, CD44, and the level of phosphorylated PI3K and AKT caused by IFIT3 knockdown in FaDu and Cal27 cells. As expected, these molecules expression levels induced by IFIT3 over-expression were also reversed by PD-L1 knockdown (Fig. 7E and F). These results suggested that IFIT3 enhanced PI3K-AKT signaling pathway to promote CSCs characteristics and EMT by interacting with PD-L1 in HNSC.

### IFIT3 promoted tumor growth and lung metastasis in nude mice

**Fig. 8:**
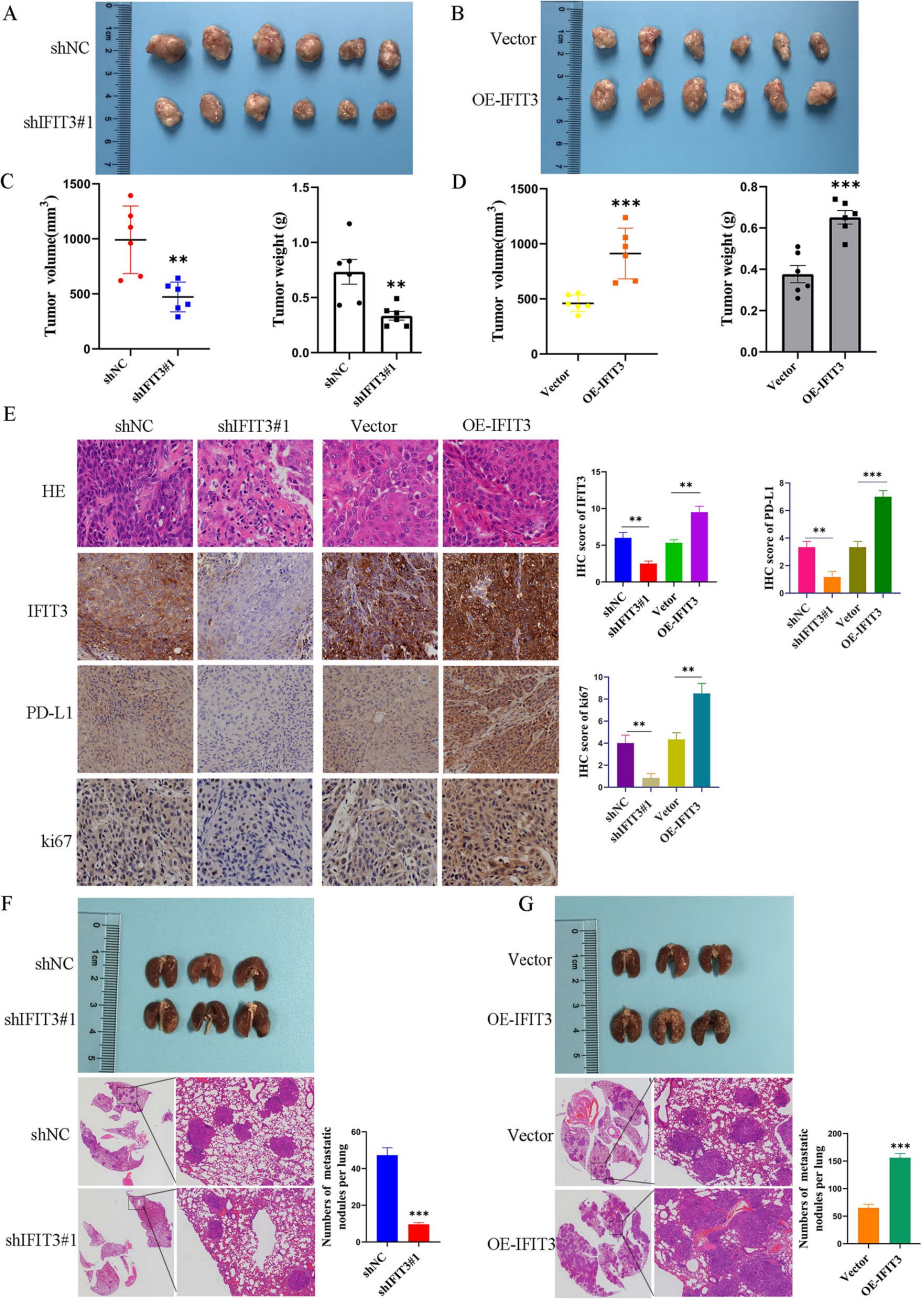
IFIT3 promoted tumorigenicity and the metastasis of HNSC in nude mice. **A**–**D** Xenograft tumor volume and weight of the xenograft tumors were measured after mice were sacrificed, IFIT3 knockdown suppressed tumor growth (**A**, **C**). IFIT3 upregulation enhanced tumor growth (**B**, **D**). **E** IHC analysis of IFT3 and PD-L1 expression in xenograft tumor tissues. **F** IFIT3 knockdown suppressed numbers of lung metastatic nodules stained with H&E. **G**. IFIT3 over-expression promoted numbers of lung metastatic nodules stained with H&E

To verify the role of IFIT3 in vivo, the model was constructed using Cal27 cells with knockdown and over-expressed IFIT3. As shown in Fig. 8A–D, IFIT3 knockdown markedly inhibited the growth of tumor volume and decreased tumor weigh in xenograft nude mice model compared to the control, and the growth of xenograft tumors was significantly promoted by IFIT3 upregulation. Furthermore, the expression levels of IFIT3, PD-L1, and ki67 were confirmed by IHC staining. PD-L1 expression and ki67 expression were decreased in IFIT3 knockdown group, and PD-L1 expression and ki67 expression were increased in IFIT3 over-expressed group (Fig. 8E). However, we established metastatic model with Cal27 cells injection via mouse tail veins. In H&E staining, the number of lung metastasis nodules in IFIT3 knockdown group was reduced, while IFIT3 over-expression markedly increased lung metastasis nodules in vivo (Fig. 8F and G). Collectively, these data indicated that IFIT3 promoted tumorigenesis and metastasis of HNSC in vivo.

## Discussion

HNSC is a common malignant tumor with a poor prognosis characterized by rapid growth and a high metastasis rate over the world [16]. Currently, although surgery is an effective treatment for locally advanced HNSC tumors, there are currently few options available for those that have disseminated; the clinical treatment of patients with metastatic HNSC remains a major clinical challenge because of heterogeneity [17]. Tumorigenesis of HNSC is a multistep, multistage process involving multiple genes; therefore, further exploration of the molecular mechanism of HNSC progression is urgently required. IFIT3 is a member of the IFIT family, previous investigations have shown that IFIT3 plays an important role in response to virus and immune system process, and the current understanding is limited to the antiviral immune response and innate immunity [18, 19]. Biological and oncogenic functions of other IFIT family members have been demonstrated, and IFIT1 increased pancreatic cancer cell proliferation, migration, and invasion by activating the Wnt/β-catenin pathway [20]. In CRC, IFIT1 served as a potential oncogene and regulated PD-L1 protein levels through altering PD-L1 ubiquitination [21]. In addition, IFIT1 was over-expressed in HNSC and indicated poor prognoses for patients with HNSC [22]. Recent studies of IFIT3 have highlighted its function as oncogene in various human cancers including breast cancer, pancreatic cancer, and liver cancer, and high expression of IFIT3 enhanced chemotherapy resistance of PDAC cells and was independently correlated to shorter patients’ survival [23]. However, the function and underlying carcinogenic mechanisms of IFIT3 in HNSC are particularly required further exploration.

In this study, we reported that the expression level of IFIT3 in HNSC was increased in TCGA and GEO databases, which was confirmed by RT-PCR and Western blotting analysis in the HNSC fresh tissues than that in adjacent normal tissues. It was interesting to note that IHC analysis showed aberrant up-regulation of IFIT3 in HNSC tissue microarray and positively correlated with advanced clinical tumor stages and lymph node metastasis respectively. Therefore, IFIT3 may serve as an oncogene and participate in HNSC carcinogenesis and progression, consistent with a previous study in other cancer.

EMT is a process associated with tumor malignant progression such as invasion and metastasis resulting in the loss of cell–cell polarity and adhesion and often controlled by multiple signaling pathways [24]. It was demonstrated that IFIT5 could induce EMT, promote cell migration and invasion, and increase the expression of ICAM1 in BCa via downregulation of mature miR-99a [25]. Study had also shown that IFIT5 promoted EMT leading to renal cancer invasion [7]. Recent studies have shown that the EMT is significantly associated with various invasive behaviors in HNSC [26], but the relationship between EMT and IFIT3 expression level remains to be further explored. In this study, we found that IFIT3 suppressed the expression of the epithelial marker, E-cadherin, while promoted the expression of the mesenchymal indicators, N-cadherin and vimentin, and we confirmed that downregulation of IFIT3 resulted in a decreased proliferation, migration, and invasion of HNSC cells. On the contrary, over-expression of IFIT3 induced EMT events in HNSC, indicating the developing process of malignant cellular behavior. Studies show that cancer cells that undergo EMT have CSC characteristics [27, 28]. CD44 is a marker of HNSC CSCs. Our findings showed that IFIT3 over-expression enhanced the number of CD44^+^ cells in HNSC, as well as the protein levels of CD44 in HNSC cells. Additionally, IFIT3 promoted the formation of tumor spheroids and enhanced HNSC cells’ capacity to start tumors.

The PI3K/Akt signaling pathway plays an important role in the process of EMT and has attracted widespread attention as a potential target for the prevention and treatment of metastatic tumors [29]. In our research, we further investigated expression levels of PI3K/AKT pathway. Compared with the control group, the phosphorylation levels of PI3K and AKT were significantly reduced after IFIT3 knockdown, while total PI3K and AKT levels had no change. The opposite trend was shown in the IFIT3 over-expressed groups. PD-L1 could crucially contribute to the maintenance of CSC self-renewal and influences cell spreading, migration, and invasion in cancer [30, 31]. In our research, we confirmed that IFIT3 and PD-L1 interact with each other, and knockdown and over-expressed PD-L1 reversed these phenotypes which were caused by IFIT3. The alterations of PD-L1 expression levels also reversed the IFIT3-induced PI3K/AKT pathway activation. Meanwhile, IFIT3 over-expression promoted tumor growth and metastasis of HNSC in vivo.

## Conclusion

In conclusion, our findings demonstrate that IFIT3 is up-regulated in HNSC; IFIT3 facilitates EMT process and CSCs properties, and promotes proliferation, migration, and invasion by targeting PD-L1 to activate the PI3K/AKT pathway. Moreover, we propose that targeting of IFIT3 may serve as a potential therapeutic strategy for patients with HNSC.

### Supplementary Information

**Additional file 1: Supplementary Table 1.** The shRNA sequences. Primers for RT-PCR.

**Additional file 2: Supplementary Table 2.** List of antibodies and suppliers used for western blot.

## Data Availability

The datasets used and/or analyzed during the present study are available from the corresponding author on reasonable request.

## Abbreviations

HNSC: Head and neck squamous cell carcinoma
TCGA: The Cancer Genome Atlas
GEO: Gene Expression Omnibus
IFIT3: Interferon-induced protein with tetratricopeptide repeats 3
EMT: Epithelial-to-mesenchymal transition
CSCs: Cancer stem cells
LN: Lymph node
PD-L1: Programmed death-ligand 1
CCK-8: Cell Counting Kit-8 assay
IHC: Immunohistochemical staining
RT-PCR: Quantitative real-time PCR
IF: Immunofluorescence assay
Co-IP: Co-immunoprecipitation
