# Supplementary material for: IFIT3 accelerates the progression of head and neck squamous cell carcinoma by targeting PD-L1 to activate PI3K/AKT signaling pathway
Source: World J Surg Oncol. 2024 Jan 25;22:34. doi: 10.1186/s12957-023-03274-5 (PMC10809513; doi:10.1186/s12957-023-03274-5)
Supplement: Supplementary file 1 — Additional file 1: Supplementary Table 1. The shRNA sequences. Primers for RT-PCR. [file 12957_2023_3274_MOESM1_ESM.docx]

Supplementary Table 1 The shRNA sequences

| Gene | Targeting sequences (5’-3’) |
| --- | --- |
| shNC | TTCTCCGAACGTGTCACGT |
| shIFIT3#1 | GGTTCTCTTGGGCCTGAAACT |
| shIFIT3#2 | GACTCTCAGATGCTCAGATTT |
| shcon | GCTTCGCGCCGTAGTCTTA |
| shPD-L1 | CCTGTTGTGATAACCACTATT |

Supplementary Table 1 Primers for RT-PCR

| Gene | Primers (5’-3’) |
| --- | --- |
| IFIT3 | F: CGTCAAGGTTCTCTTGGG |
|  | R: CGGAGGACATCTGTTTGG |
| GAPDH | F: CAGGAGGCATTGCTGATGAT |
|  | R: GAAGGCTGGGGCTCATTT |

Abbreviations: F, forward primer; R, reverse primer.
