# Supplementary material for: IFIT3 accelerates the progression of head and neck squamous cell carcinoma by targeting PD-L1 to activate PI3K/AKT signaling pathway
Source: World J Surg Oncol. 2024 Jan 25;22:34. doi: 10.1186/s12957-023-03274-5 (PMC10809513; doi:10.1186/s12957-023-03274-5)
Supplement: Supplementary file 2 — Additional file 2: Supplementary Table 2. List of antibodies and suppliers used for western blot. [file 12957_2023_3274_MOESM2_ESM.docx]

Supplementary Table 2 List of antibodies and suppliers used for western blot

| Antibody | Isotype | Suppliers |
| --- | --- | --- |
| IFIT3 | Rabbit IgG | CST |
| E-cadherin | Rabbit IgG | Affinity |
| N-cadherin | Rabbit IgG | Affinity |
| Vimentin | mouse IgG | UltraMAB |
| phosphorylated PI3K p85 | Rabbit IgG | CST |
| PI3K p85 | Rabbit IgG | CST |
| phosphorylated AKT | Rabbit IgG | CST |
| AKT | Rabbit IgG | CST |
| PD-L1 | Rabbit IgG | CST |
| CD44 | Rabbit IgG | Affinity |
| GAPDH | mouse IgG | ZSGB-BIO |
